# Development of a neural network for diagnosing the risk of depression according to the experimental data of the stop signal paradigm

**DOI:** 10.18699/VJGB-22-93

**Published:** 2022-12

**Authors:** M.O. Zelenskih, A.E. Saprygin, P.D. Rudych, D.A. Lebedkin, A.N. Savostyanov

**Affiliations:** Novosibirsk State University, Novosibirsk, Russia; Institute of Cytology and Genetics of the Siberian Branch of the Russian Academy of Sciences, Novosibirsk, Russia Scientific Research Institute of Neurosciences and Medicine, Novosibirsk, Russia; Novosibirsk State University, Novosibirsk, Russia Scientific Research Institute of Neurosciences and Medicine, Novosibirsk, Russia Federal Research Center of Fundamental and Translational Medicine, Novosibirsk, Russia; Novosibirsk State University, Novosibirsk, Russia Federal Research Center of Fundamental and Translational Medicine, Novosibirsk, Russia; Novosibirsk State University, Novosibirsk, Russia Institute of Cytology and Genetics of the Siberian Branch of the Russian Academy of Sciences, Novosibirsk, Russia Scientific Research Institute of Neurosciences and Medicine, Novosibirsk, Russia Federal Research Center of Fundamental and Translational Medicine, Novosibirsk, Russia

**Keywords:** stop signal paradigm, artificial neural network, system for depression, risk assessment, machine learning, стоп-сигнал парадигма, искусственная нейронная сеть, система тестирования, риск возникновения депрессии, машинное обучение

## Abstract

These days, the ability to predict the result of the development of the system is the guarantee of the successful functioning of the system. Improving the quality and volume of information, complicating its presentation, the need to detect hidden connections makes it ineffective, and most often impossible, to use classical statistical forecasting methods. Among the various forecasting methods, methods based on the use of artificial neural networks occupy a special place. The main objective of our work is to create a neural network that predicts the risk of depression in a person using data obtained using a motor control performance testing system. The stop-signal paradigm (SSP) is an experimental technique to assess a person’s ability to activate deliberate movements or inhibit movements that have become inadequate to external conditions. In modern medicine, the SSP is most commonly used to diagnose movement disorders such as Parkinson’s disease or the effects of stroke. We hypothesized that SSP could serve as a basis for detecting the risk of affective diseases, including depression. The neural network we are developing is supposed to combine such behavioral indicators as: the amount of missed responses, amount of correct responses, average time, the amount of correct inhibition of movements after stop-signal onset. Such a combination of indicators will provide increased accuracy in predicting the presence of depression in a person. The artificial neural network implemented in the work allows diagnosing the risk of depression on the basis of the data obtained in the stop-signal task. An architecture was developed and a system was implemented for testing motor control indicators in humans, then it was tested in real experiments. A comparison of neural network technologies and methods of mathematical statistics was carried out. A neural network was implemented to diagnose the risk of depression using stop-signal paradigm data. The efficiency of the neural network (in terms of accuracy) was demonstrated on data with an expert assessment for the presence of depression and data from the motor control testing system.

## Abstract (ru)

В настоящее время возможность спрогнозировать результат развития системы – залог успешного функционирования системы. Повышение качества и объема информации, усложнение ее представления, необходимость обнаруживать скрытые связи делают неэффективным, а чаще всего невозможным, применение классических статистических методов прогнозирования. Среди разнообразных методов прогнозирования особое место занимают методы, основанные на использовании искусственных нейронных сетей. Задачей нашей работы является создание нейронной сети, прогнозирующей риск возникновения депрессии у человека, с помощью данных, полученных при использовании системы тестирования показателей моторного контроля. Стоп-сигнал парадигма (ССП) – это экспериментальный метод, позволяющий оценить способность человека активировать целенаправленные движения или подавлять движения, ставшие неадекватными внешним условиям. В современной медицине ССП чаще всего при- меняется для диагностики двигательных нарушений, таких как болезнь Паркинсона или последствия инсульта. Мы предположили, что ССП может служить основой для выявления риска развития аффективных заболеваний, вклю- чая депрессию. В разрабатываемой нами нейронной сети предполагается комбинирование таких поведенческих показателей, как количество пропущенных ответов, количество правильных ответов, среднее время, количество верных торможений после появления стоп-сигнала. Такой набор показателей обеспечит повышенную точность про- гнозирования наличия депрессии у человека. Реализованная в работе искусственная нейронная сеть позволяет по данным, полученным с помощью фиксации реакции на стимулы со стоп-сигналом, диагностировать риск возникно- вения депрессии. Разработана архитектура и реализована система тестирования показателей моторного контроля у человека, затем протестирована в реальных экспериментах. Проведено сравнение нейросетевых технологий и методов математической статистики. Реализована нейронная сеть для диагностирования риска возникновения де- прессии по данным ССП. На примере данных с экспертной оценкой на наличие депрессии и результатов, получен- ных при использовании системы тестирования показателей моторного контроля, продемонстрирована эффектив- ность нейронной сети (с точки зрения точности).

## Introduction

The ability to predict the result of the development of the system is the key to the successful functioning of the system. Improving the quality and volume of information, complicating its presentation, and the need to detect hidden connections makes it ineffective, and most often impossible, to use classical statistical forecasting methods. Among the various forecasting methods, methods based on the use of artificial neural networks occupy a special place.

The main objective of our work is to create a neural network that predicts the risk of depression in a person using data obtained using the motor control indicators testing system (Haykin, 2006). All data are taken from the open database of the Institute of Cytology and Genetics of the Siberian Branch of the Russian Academy of Sciences (ICBrainDB dataset https://icbraindb.cytogen.ru/api-v2).

A group of patients with depression was examined at the clinic of the Scientific Research Institute of Neurosciences and Medicine. The presence of major depressive disorder was diagnosed by a psychiatrist during a closed interview based on The International Statistical Classification of Diseases and Related Health Problems, 10th revision (ICD-10) criteria. As a control group of healthy people, participants who had never been treated in psychiatric clinics and had not turned to psychiatrists for medical help were invited. All participants in the control group denied having any neurological or psychiatric diseases at the time of the examination or for five years before the examination. In addition, all the survey participants, both patients and control participants, denied the presence of alcohol or drug addiction and the usage of other psychoactive substances.

The main differences between artificial neural networks and methods of mathematical statistics are parallel processing of information and the ability to learn without a teacher, in other words, to self-study (https://wiki.loginom.ru/articles/ normalization.html). Below, in the form of a table (Table 1), the results of comparing neural networks and methods of mathematical statistics according to the selected criteria are presented.

**Table 1.**
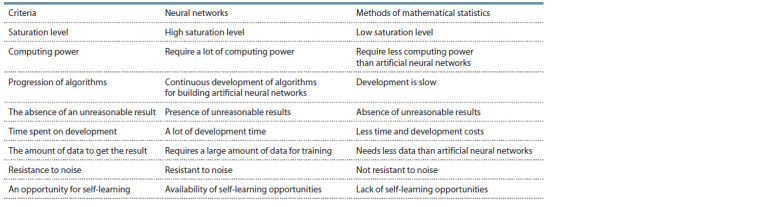
Comparison of neural networks and mathematical statistics

Resistance to noise is an important indicator when working with a large number of parameters and at the absence of explicit dependencies that we get from the data of the stop signal paradigm. Self-study makes it possible to perform tasks without outside interference, which contributes to the search for patterns between parameters.

The use of mathematical statistics methods in the search for dependencies between the stop signal paradigm and the risks of depression cannot fully detect their presence due to the sensitivity of the methods to superfluous data, and even more so they cannot further predict the risk of depression in a person. Noise resistance and self-learning make usage of neural networks not simply preferable, compared to mathematical statistics, but necessary.

The neural network should accept a dataset consisting of data obtained using the stop signal paradigm as input and output the diagnostic result for the risk of depression.

The stop signal paradigm (SSP) is an experimental method that allows us to evaluate a person’s ability to activate deliberate movements or suppress movements that have become inadequate to external conditions. In modern medicine, SSP is most often used to diagnose motor disorders, such as Parkinson’s disease or the consequences of a stroke. We suggested that SSP can serve as a basis for identifying the risk of developing affective diseases, including depression. The neural network we are developing assumes a combination of behavioral indicators such as: the number of missed answers, the number of correct answers, the average time, the number of correct stops. Such a set of indicators will provide increased accuracy in predicting the presence of depression in a person.

The purpose of this work is to develop a neural network for predicting the risk of depression according to the stop signal paradigm. The artificial neural network implemented in the work makes it possible to predict the risk of depression based on the data obtained by registering the reaction to stimuli with a stop signal.

## Materials and methods

Implementation of a neural network. The following table shows the technologies used for implementation along with a rationale (Table 2).

**Table 2.**
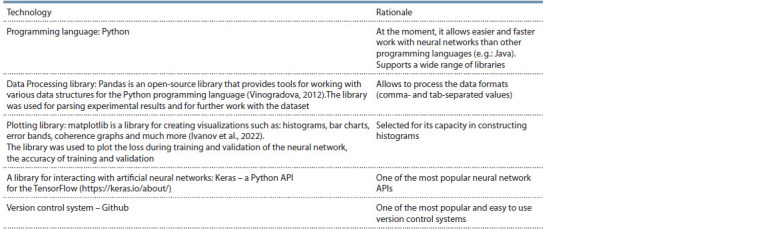
Technology stack used

The architecture of the model. To work with the model and layers, the Sequential and Dense classes of the Tensor- Flow were used.

The Sequential class is a sequential neural network architecture, which is equivalent to sequential layer invocation (https://keras.io/api/layers/core_layers/dense/).

The Dense class implements the operation: output = activation(dot(input, kernel) + bias), (1) where activation is the element-by-element activation function passed as an argument, kernel – is the matrix of all weights created by the layer, bias – is the displacement vector created by the layer (https://keras.io/api/layers/activations/). Two layers were highlighted: – layer x, that is, a layer for working with objects based on input data with the exception of the category of the test; – layer y, that is, a layer for working with answers based on the category of the test subject.

To work with layer x, the relu activation function was used. The relu function returns a number if it accepts a positive argument, in other cases it returns 0 (https://matplotlib.org/ stable/index.html). To work with layer y, the sigmoid activation function was used, which is necessary for probabilistic forecasting. Sigmoid activation function: sigmoid(x) = 1 (1 + exp(–x)). (2) For small values, the function returns a value close to 0, and for large values, it returns close to 1, and the sigmoid always returns from 0 to 1 (https://www.probabilitycourse.com/ chapter9/9_1_5_mean_squared_error_MSE.php).

Data collection for training. In preparation for the development of the neural network, a balanced dataset was created based on data obtained during the examination of healthy people and patients with diagnosed depression. The following inputs were highlighted: – Missed – the number of missed responses from the test subject; – Right – the total number of correct answers from the test subject; – Av_time – average reaction time for the test subject during the experiment; – Stop – the number of correct ignores on the stop signal of the test subject; – Practice – the number of correct answers in the block “Practice” at the test; – Right_stop – the number of correct answers without taking into account the stop signal; – Incor_stop – the number of incorrect reactions to the stop signal; – Survive – the category of the test subject (healthy or diagnosed with depression).

Data preparation and normalization. Data normalization is a procedure for preprocessing input data, in which the values of the features forming the input vector are reduced to a specified range. Normalization is necessary because the initial values can vary over a large range and the operation of a neural network with such data can lead to an incorrect result (https://keras.io/api/models/). Normalization of data to the range [0...1] is important for setting a single privilege of features, in other words, for setting the same significance for each feature, which will allow them to be compared with each other in equal conditions.

All dataset inputs were selected for normalization, with the exception of Survive, since this parameter is an estimate and takes only two values: 0 or 1.

Network topology selection. Choosing the topology of an artificial neural network is one of the most important stages when using neural network technologies to solve practical problems. The adequacy of neural network model training directly depends on this stage (https://keras.io/api/models/ model_training_apis/). Since we are faced with the task of classification and it is important to find any hidden connections, we need each artificial neuron to be connected to other neurons.

Based on the concepts of neural network types, a fully connected type was chosen, since, as mentioned earlier, each artificial neuron transmits its output to the rest of the neurons.

Experimental selection of training parameters. During this stage of neural network development, it is necessary to select optimal training parameters that will demonstrate the best accuracy and loss indicators. Selection is carried out by launching a neural network with possible parameters and a test dataset.

The following table (Table 3) shows the results of the experimental selection of training parameters, that is, the selection of the number of passes of the dataset from beginning to end (epochs) and the amount of data for validation (validation_ split) on a balanced dataset (50 % healthy, 50 % with diagnosed depression, total 205).

**Table 3.**
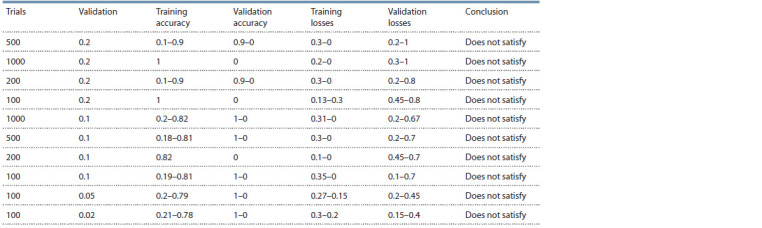
Selection of parameters on a balanced dataset

**Fig. 1.**
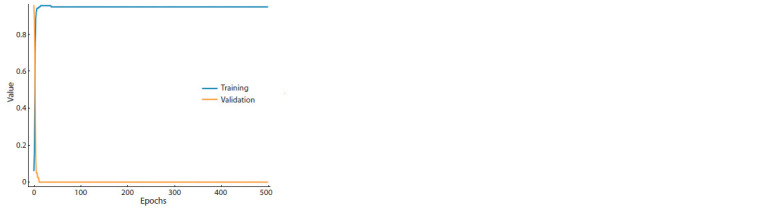
An example of a graph of training accuracy and validation accuracy when training on a balanced dataset

Figure 1 demonstrates the accuracy of training and validation when training on a balanced dataset with a choice of epochs = 500 and validation_split = 0.2.

Thus, due to the lack of suitable parameters for further work, it was decided to use an unbalanced dataset (65 % of healthy, 35 % with diagnosed depression, only 500).

The following table shows the results of experimental selection of training parameters on an unbalanced dataset (Table 4).

**Table 4.**
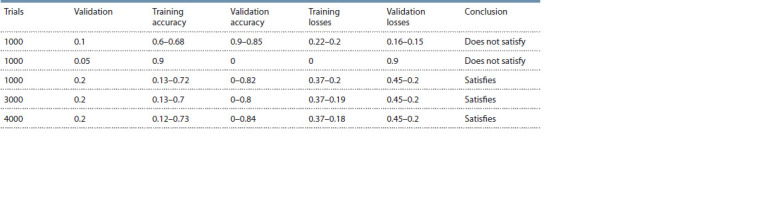
Selection of parameters on an unbalanced dataset

**Fig. 2.**
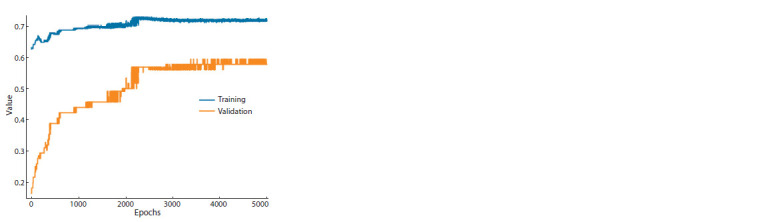
An example of a graph of training accuracy and validation accuracy when training on an unbalanced dataset.

Figure 2 demonstrates the accuracy of training and validation when training on an unbalanced dataset with epochs = = 5000 and validation_split = 0.2.

Based on the results obtained, the number of passes from the beginning of the dataset to the end (epoch) = 4000 was selected, the amount of data for validation (validation_split) = = 0.2.

Neural network training. To ensure the correctness of the artificial neural network, the sample was divided into two parts: training data for training, verification data for checking the operation of the neural network.

The compile and fit methods were used for training. The arguments of the compile method are: optimizer, loss function, metrics, loss weights, list of metrics. In the fit method, the arguments are: input data, target data, number of samples, number of epochs, list of callbacks, amount of data for validation (https://pandas.pydata.org/pandas-docs/stable/).

Arguments used in the compile method: • loss = “mse” – root-mean-square error: E[(X – X )2] = E[(X – g(Y ))2], (3) let X = g(Y ) be an estimate of a random variable, given the observation of a random variable Y (https://www.journaldev.com/45330/relu-function-in-python); • optimizer = “sgd” – gradient descent optimizer taking into account momentum (https://keras.io/api/optimizers/sgd/); • metrics = [“accuracy”]. Arguments used in the fit method: • x – input data; • y – the target data, that is, the estimate; • epochs = “4000” – the number of epochs; • validation_split = “0.2” – the amount of validation data used in the training sample.

Checking the adequacy of training. Testing of the adequacy of training is carried out on data that were not in the training samples, in other words, new data for the neural network are used.

The following table (Table 5) shows an example of a sequence of values (PSurvived) obtained from the neural network, taking into account the category of data.

**Table 5.**
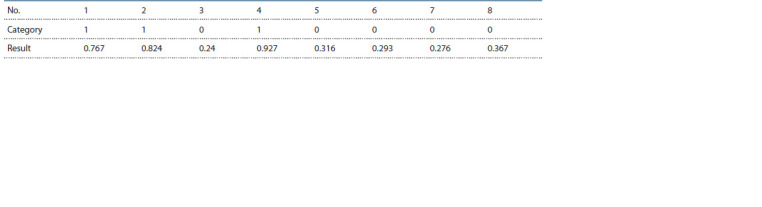
Checking the adequacy of training Notе. 0 – with diagnosed depression, 1 – without depression

## Results

Technical tests. For technical tests of the neural network, data from experiments on our system for testing human motor control indicators (without expert assessment for depression, that is, without clinical confirmation) were used, as well as previously unused data that did not participate in the training sample (with expert assessment).

The purpose of the technical tests is to study how the developed neural network will cope with the classification for the presence of risks of depression according to the stop signal paradigm

Input data. The following input data were selected for the technical tests of the neural network: • Unbalanced dataset (0.37 – with diagnosed depression, 0.63 – without depression); • The maximum number of missed responses is 85; • The maximum total number of correct answers for the test – 92; • The maximum average time per experiment for a test subject is 750.0; • The maximum number of correct ignores for a stop signal from a test subject is 34; • The maximum number of correct answers in the “Practice” block in the test – 31; • The maximum number of correct answers without taking into account the stop signal is 65; • The maximum number of incorrect reactions to the stop signal is 35; • The amount of data for validation is 0.2, the number of epochs is 4000.

Test results. The following table describes the results of the neural network with an estimate of the values obtained (Table 6).

**Table 6.**
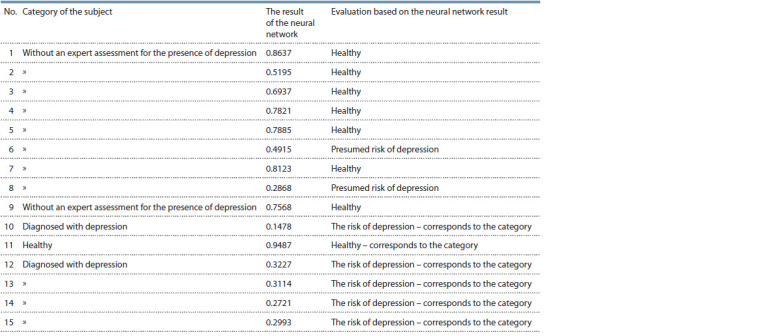
The results of the neural network

Thus, during the technical tests, the results of the neural network were obtained, which demonstrate which category (healthy/at risk of depression) the test subject belongs to. The obtained indicators fully correspond to the diagnoses.

## Conclusion

Based on the experimental data obtained using the stop signal paradigm, a dataset was formed. The implementation of a neural network for diagnosing the risk of depression according to the stop signal paradigm has been developed and further tested. Using the example of data with an expert assessment for the presence of depression and data obtained using the motor control indicators testing system, the accuracy of the neural network classification was shown. The test results in the form of performance indicators of the neural network are described below: Indicator Meaning Training losses 0.1657 Training accuracy 0.7821 Validation losses 0.2415 Validation accuracy 0.6667

The stop signal paradigm is commonly used to diagnose motor disorders such as Parkinson’s disease, childhood hyperactivity or post-traumatic disorders. Previously, the stop signal paradigm was not used by anyone to diagnose depression. We applied this technique in combination with neural network methods and showed that the results of SSP make it possible to efficiently classify people into patients with depression and people without depression. It should also be noted that we did not compare patients with depression with patients with other non-depression-related neurological diseases. Therefore, at the moment, it is not yet clear whether our method allows us to divide patients with different disorders into different subclasses.

## Conflict of interest

The authors declare no conflict of interest.
